# Supplementary material for: Identification of distinct cDC2 subpopulations that direct microbiota-specific T cell differentiation
Source: bioRxiv. 2025 Nov 5:2025.11.04.686414. Preprint. [Version 1] doi: 10.1101/2025.11.04.686414 (PMC12637724; doi:10.1101/2025.11.04.686414)
Supplement: Supplement 6 [file NIHPP2025.11.04.686414v1-supplement-6.pdf]

bioRxiv preprint doi: https://doi.org/10.1101/2025.11.04.686414; this version posted November 5, 2025. The copyright holder for this preprint (which was not certified by peer review) is the author/funder, who has granted bioRxiv a license to display the preprint in perpetuity. It is made available under aCC-BY 4.0 International license.

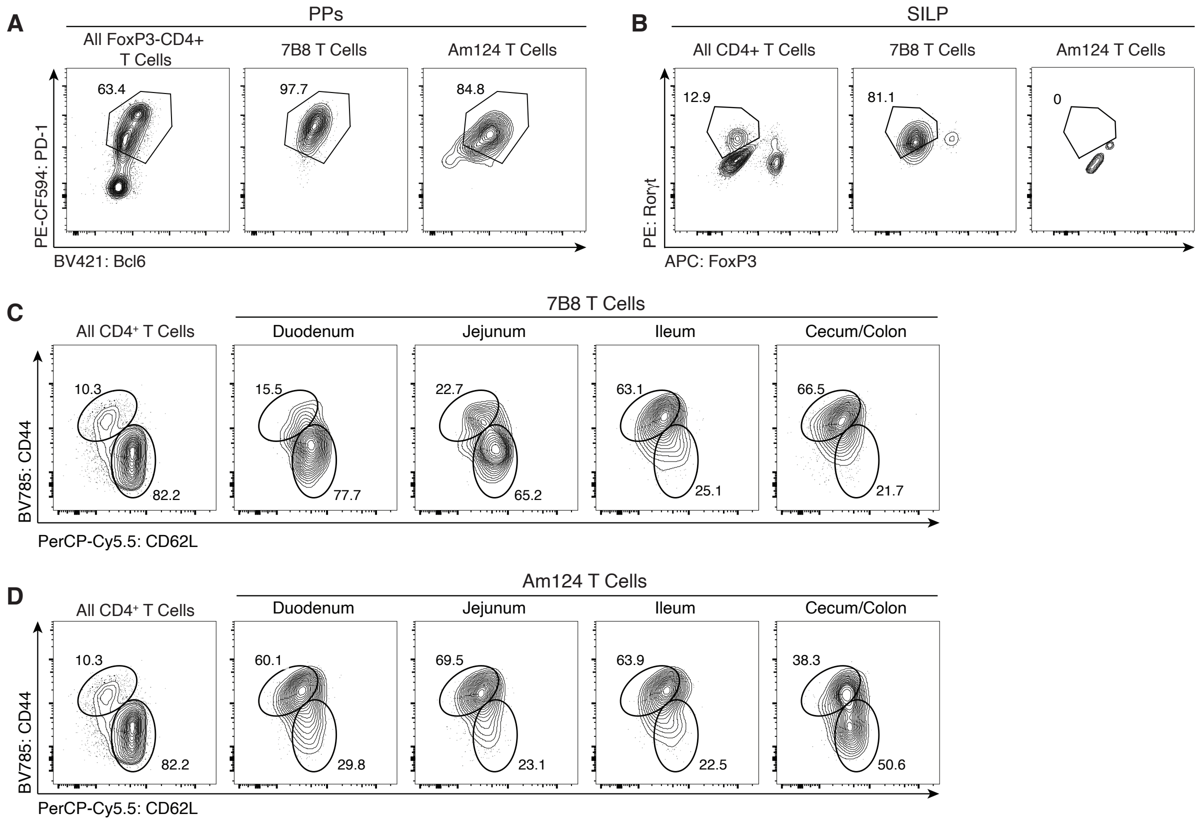

Supplementary Figure 1: Expression of differentiation and activation markers by 7B8 and Am124 T cells. (**A**) Representative flow plots showing expression of T_{FH} markers (Bcl6 and PD-1) by endogenous (left), 7B8 (center), and Am124 (right) T cells in the PPs of ASF+Akk+SFB mice. (**B**) Representative flow plots showing expression of $T_{H}17$ markers (Rorγt^{+} FoxP3^{-}) by endogenous (left), 7B8 (center), and Am124 (right) in the SILP of ASF+Akk+SFB mice. (**C**) Representative flow plots showing activated (CD44^{+} CD62L^{-}) 7B8 T cells in segmented gLNs. (**D**) Representative flow plots showing activated (CD44^{+} CD62L^{-}) Am124 T cells in segmented gLNs.

bioRxiv preprint doi: https://doi.org/10.1101/2025.11.04.686414; this version posted November 5, 2025. The copyright holder for this preprint (which was not certified by peer review) is the author/funder, who has granted bioRxiv a license to display the preprint in perpetuity. It is made available under aCC-BY 4.0 International license.

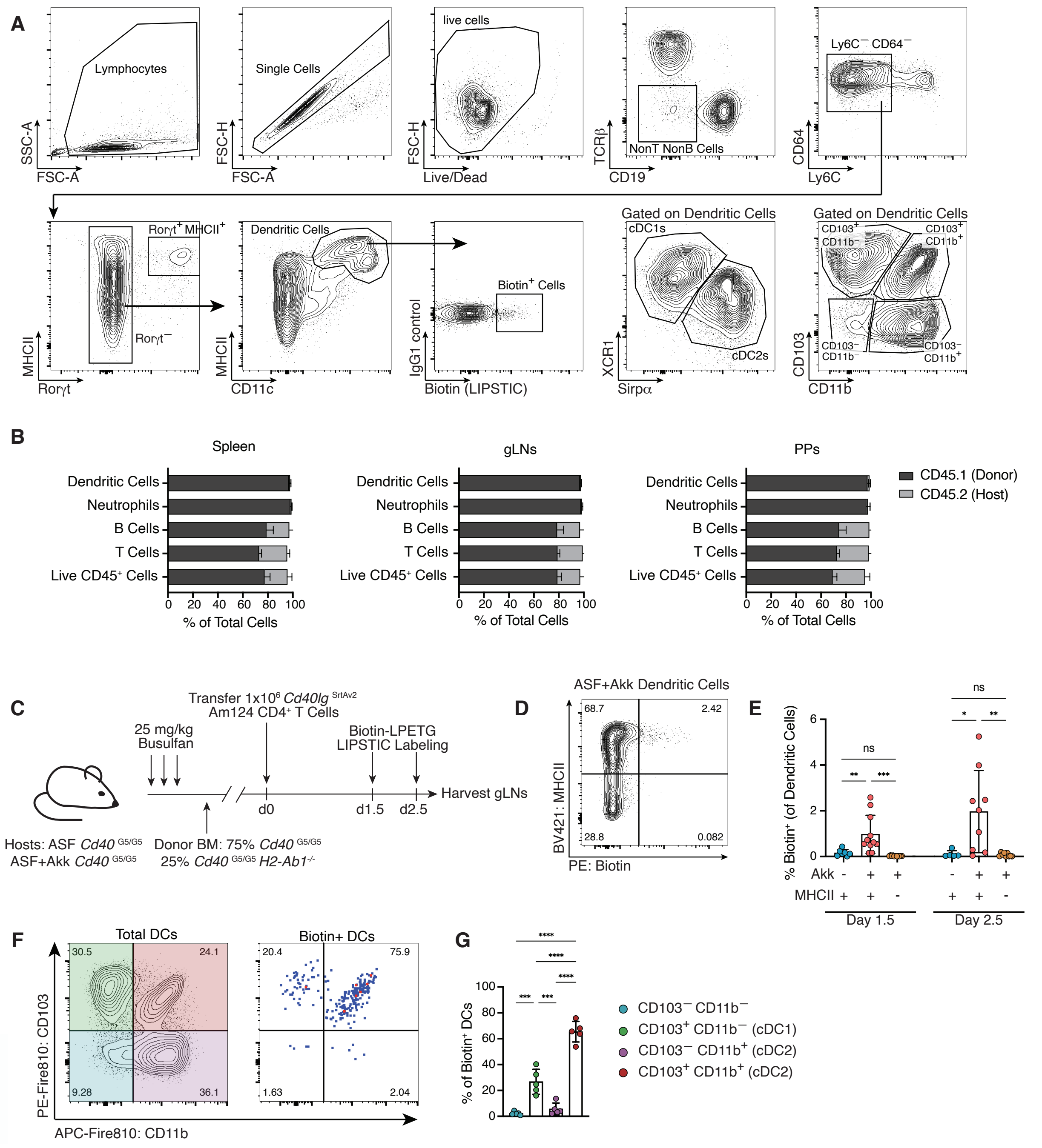

Supplementary Figure 2: Additional characterization of the Am124 LIPSTIC-labeling system. (**A**) Flow plots showing the general gating scheme for cDCs. (**B**) C57BL/6N (CD45.2$^+$) mice were treated with 25 mg/kg busulfan every day for three days (75 mg/kg total), followed by reconstitution with JAXBoy (CD45.1$^+$) bone marrow. Spleen (left), gLNs (middle) and PPs (right) were harvested 8 to 12 weeks later. Plots show frequency of CD45.2$^+$ (host) and CD45.1$^+$ (donor) cells out of total live cells, T cells, B cells, neutrophils, and cDCs. Error bar represents mean and standard deviation (n = 5 mice per group, data is representative of two independent experiments). (**C**) Experimental timeline for (**D** and **E**). BMCs were generated by treating mice

bioRxiv preprint doi: https://doi.org/10.1101/2025.11.04.686414; this version posted November 5, 2025. The copyright holder for this preprint (which was not certified by peer review) is the author/funder, who has granted bioRxiv a license to display the preprint in perpetuity. It is made available under aCC-BY 4.0 International license.

with 25 mg/kg busulfan daily for three days (75 mg/kg total), followed by reconstitution with 75% *Cd40*$^{G5/G5}$ bone marrow and 25% *Cd40*$^{G5/G5}$ *H2-Ab1*$^{-/-}$ bone marrow. After 8 to 12 weeks, 1x 10^6 *Cd40lg*^{SrtAv2} Am124 T cells were adoptively transferred and LIPSTIC labeling was performed 1.5 and 2.5 days post transfer. (**D**) Representative flow plot showing LIPSTIC labeling of MHCII$^+$ and MHCII$^–$ cDCs in mixed BMCs. cDCs were gated as Live Thy1.2$^–$ F4/80$^–$ SiglecF$^–$ Ly6G$^–$ CD19$^–$ CD64$^–$ CD11c$^+$ to avoid gating with MHCII. (**E**) Frequency of labeled biotin$^+$ cDCs in the gLNs of ASF and ASF+Akk mixed BMCs at 1.5 and 2.5 days post transfer. For ASF+Akk mice, cells are divided based on the expression of MHCII. All mice received LPETG substrate. For (C to E), n = 3 to 7 mice per group, per experiment; data is pooled from two independent experiments. (**F**) Representative flow plots showing expression of CD11b and CD103 on total (left) and biotin$^+$ cDCs (right) in gLNs. (**G**) Percentage of biotin$^+$ DCs expressing CD103 and CD11b. For (F to G), n = 3 to 5 mice per group; data is representative of three independent experiments. For all plots, each symbol represents one mouse and error bars represent mean and standard deviation. P-values were calculated using one-way ANOVA. Statistical significance denoted as not significant (ns), *P < 0.05, **P < 0.01, ***P < 0.001, ****P < 0.0001.

bioRxiv preprint doi: https://doi.org/10.1101/2025.11.04.686414; this version posted November 5, 2025. The copyright holder for this preprint (which was not certified by peer review) is the author/funder, who has granted bioRxiv a license to display the preprint in perpetuity. It is made available under aCC-BY 4.0 International license.

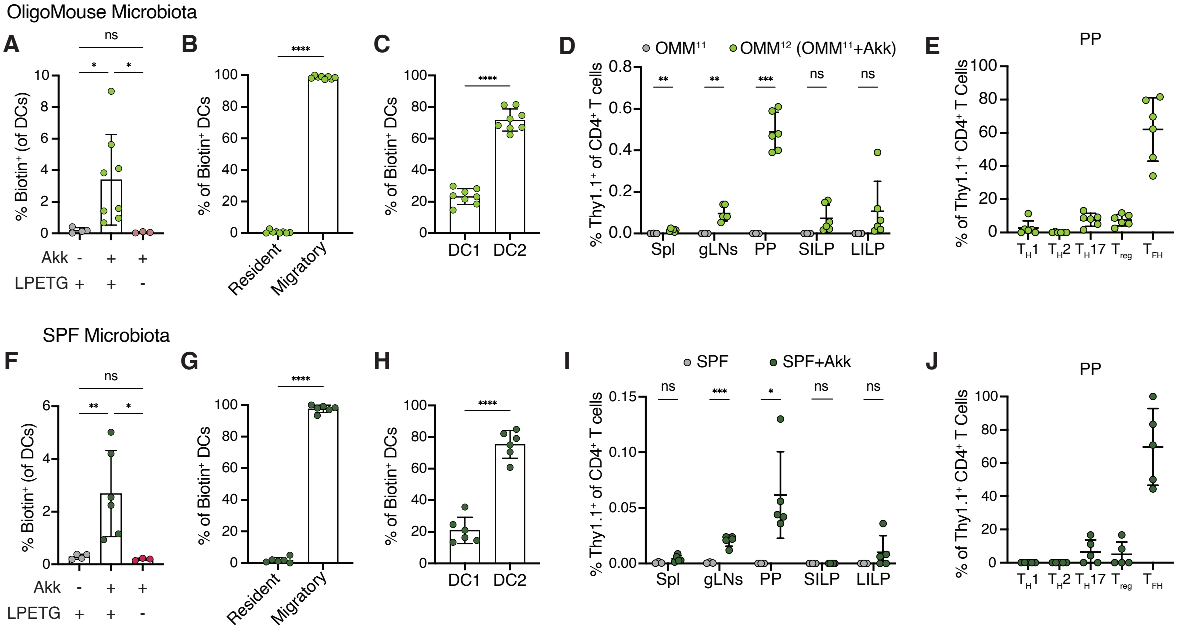

Supplementary Figure 3: *A. muciniphila*-specific T cells are primed by migratory cDC2s and differentiate into T follicular helper cells in mice with complex microbiota. For (**A** to **C**), 1x10^6 *Cd40lg*^{SrtAv2} Am124 T cells were adoptively transferred into OMM^{11} and OMM^{12} (OMM^{11}+Akk) mice and LIPSTIC labeling was performed 2.5 days later to identify *A. muciniphila*-presenting cells. (**A**) Percentage of labeled biotin$^+$ DCs in gLNs of OMM^{11} and OMM^{12} mice. (**B**) Percentage of migratory and resident cDCs out of total biotin$^+$ cDCs from (A). (**C**) Percentage of cDC1s (Xcr1$^+$) and cDC2s (Sirpα$^+$) out of total biotin$^+$ cDCs from (A). For (A to C), n = 3 to 8 mice per group, data representative of two independent experiments. For (**D** and **E**), 1x10^4 Am124 T cells were adoptively transferred into OMM^{11} and OMM^{12} mice. Spleen, gLNs, PPs, SILP, and LILP were harvested 12 days later. (**D**) Frequency of transferred Am124 T cells in tissues of OMM^{11} and OMM^{12} mice. (**E**) Expression of T_H1 (Tbet$^+$ FoxP3$^-$), T_H2 (Gata33$^+$ FoxP3$^-$), T_H17 (Rorγt$^+$ FoxP3$^-$), T_{REG} (FoxP3$^+$), and T_{FH} (Bcl6$^+$ PD-1$^+$ FoxP3$^-$) markers in transferred Am124 in the PPs of OMM^{12} mice from (D). For (D and E), n = 3 to 6 mice per group, data representative of two independent experiments. For (**F** to **H**), 1x10^6 *Cd40lg*^{SrtAv2} Am124 T cells were adoptively transferred into SPF and SPF+Akk mice and LIPSTIC labeling was performed 2.5 days later to identify *A. muciniphila*-presenting cells. (**F**) Percentage of labeled biotin$^+$ DCs in gLNs of SPF and SPF+Akk mice. (**G**) Percentage of migratory and resident cDCs out of total biotin$^+$ cDCs from (F). (**H**) Percentage of cDC1s (Xcr1$^+$) and cDC2s (Sirpα$^+$) out of total biotin$^+$ cDCs from (F). For (F to H), n = 3 to 6 mice per group, data representative of two independent experiments. For (**I** and **J**), 1x10^4 Am124 T cells were adoptively transferred into SPF and SPF+Akk mice. Spleen, gLNs, PPs, SILP, and LILP were harvested 12 days later. (**I**) Frequency of transferred Am124 T cells in tissues of SPF and SPF+Akk mice. (**J**) Expression of T_H1 (Tbet$^+$ FoxP3$^-$), T_H2 (Gata3$^+$ FoxP3$^-$), T_H17 (Rorγt$^+$ FoxP3$^-$), T_{REG} (FoxP3$^+$), and T_{FH} (Bcl6$^+$ CXCR5$^+$ FoxP3$^-$) markers in transferred Am124 in the PPs of SPF+Akk mice from (I). For (I and J), n = 3 to 5 mice per group, data representative of two independent experiments. For all graphs, each symbol represents one mouse and error bars

bioRxiv preprint doi: https://doi.org/10.1101/2025.11.04.686414; this version posted November 5, 2025. The copyright holder for this preprint (which was not certified by peer review) is the author/funder, who has granted bioRxiv a license to display the preprint in perpetuity. It is made available under aCC-BY 4.0 International license.

represent mean and standard deviation. P-values were calculated by one-way ANOVA for (A), and (F), and by unpaired T-test for (B to E) and (G to J). Statistical significance denoted as not significant (ns), *P < 0.05, **P < 0.01, ***P < 0.001, ****P < 0.0001.

bioRxiv preprint doi: https://doi.org/10.1101/2025.11.04.686414; this version posted November 5, 2025. The copyright holder for this preprint (which was not certified by peer review) is the author/funder, who has granted bioRxiv a license to display the preprint in perpetuity. It is made available under aCC-BY 4.0 International license.

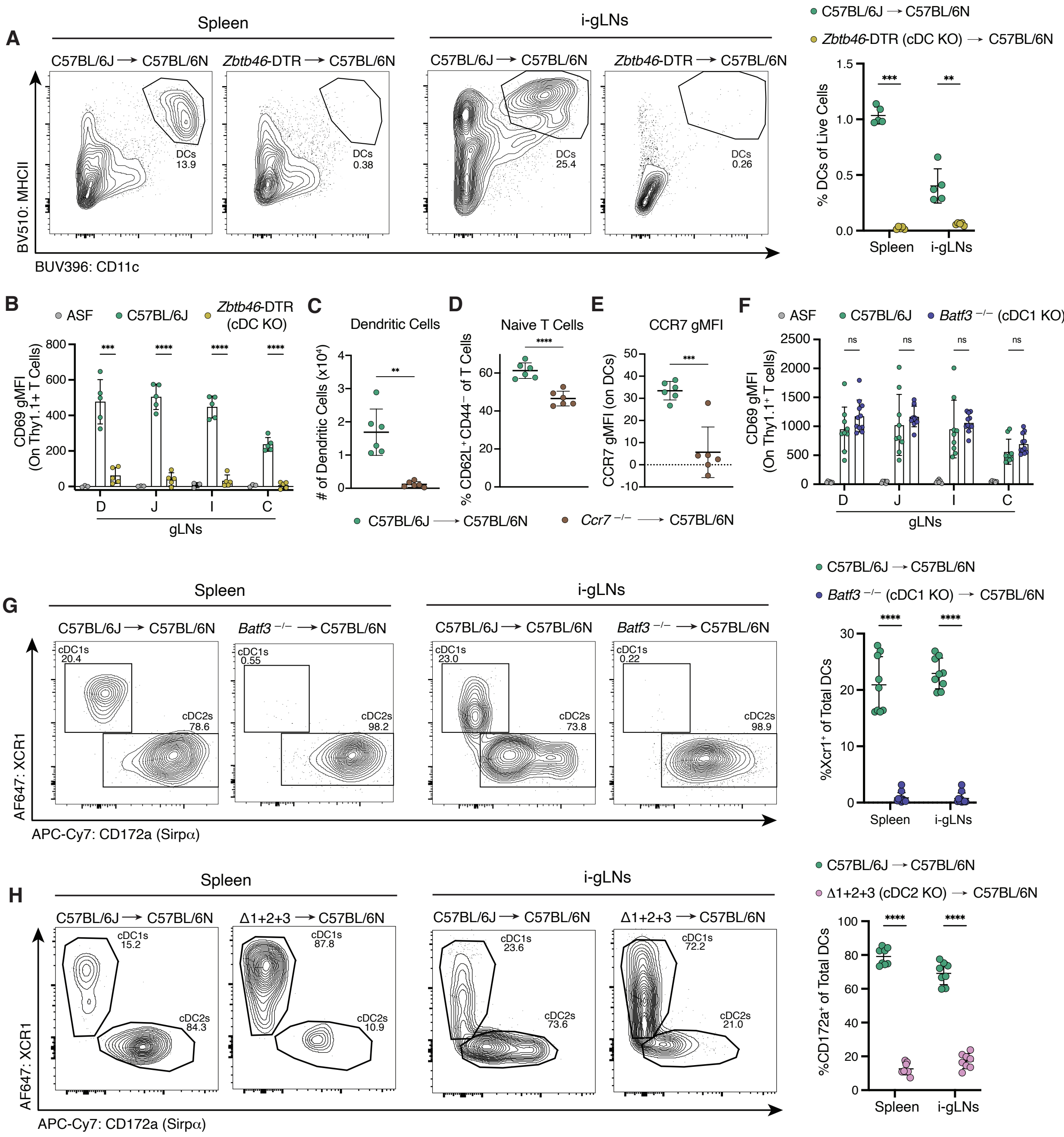

Supplementary Figure 4: Characterization of ASF+Akk busulfan BMCs. ASF+Akk mice were treated with 25 mg/kg busulfan daily for three days (75 mg/kg total) followed by reconstitution with the indicated bone marrow genotype. 8 to 12 weeks later, 1.5x10^{5}Am124 T cells were adoptively transferred into ASF control and ASF+Akk BMC mice. gLNs and spleen were harvested 1.5 days later. (**A**) Representative flow plots (left) and frequency (right) of cDCs in spleen and ileal gLNs of diphtheria-toxin-treated ASF+Akk BMCs reconstituted with C57BL/6J or *Zbtb46*-DTR bone marrow (n = 5 mice per group; data is representative of two

bioRxiv preprint doi: https://doi.org/10.1101/2025.11.04.686414; this version posted November 5, 2025. The copyright holder for this preprint (which was not certified by peer review) is the author/funder, who has granted bioRxiv a license to display the preprint in perpetuity. It is made available under aCC-BY 4.0 International license.

independent experiments). (**B**) Expression of CD69 on Am124 T cells in gLNs of ASF control mice or diphtheria-toxin-treated ASF+Akk BMCs reconstituted with C57BL/6J or *Zbtb46*-DTR bone marrow (n = 3 to 5 mice per group, data is representative of two independent experiments). (**C**) Number of DCs in the ileal gLNs of ASF+Akk BMCs reconstituted with C57BL/6J or *Ccr7*$^{-/-}$ bone marrow. (**D**) Frequency of naive (CD62L^{+}CD44^{-}) T cells in ileal gLNs of ASF+Akk BMCs reconstituted with C57BL/6J or *Ccr7*$^{-/-}$ bone marrow. (**E**) gMFI of CCR7 on DCs in ileal gLNs of ASF+Akk BMCs reconstituted with C57BL/6J or *Ccr7*$^{-/-}$ bone marrow. For (C to E), n = 6 mice per group, data is representative of two independent experiments. (**F**) Expression of CD69 on Am124 T cells 1.5 days post-transfer in gLNs of ASF control mice or ASF+Akk BMCs reconstituted with C57BL/6J or *Batf3*$^{-/-}$ bone marrow (n = 3 to 6 mice per group, per experiment, data is pooled from two independent experiments). (**G**) Representative flow plots (left) and frequency (right) of cDC1s in spleen and ileal gLNs of ASF+Akk BMCs reconstituted with C57BL/6J or *Batf3*$^{-/-}$ bone marrow (n = 4 to 6 mice per group, per experiment, data is pooled from two independent experiments). (**H**) Representative flow plots (left) and frequency (right) of cDC2s in spleen and ileal gLNs from ASF+Akk BMCs reconstituted with C57BL/6J or Δ1+2+3 bone marrow (n = 4 mice per group, per experiment, data is pooled from two independent experiments). For all graphs, each symbol represents one mouse and error bars represent mean and standard deviation. P-values were calculated by unpaired T-test with Welch's correction for (B), (C), and (F) and unpaired T-test for (A), (D), (E), (G), and (H). Statistical significance denoted as not significant (ns), *P < 0.05, **P < 0.01, ***P < 0.001, ****P < 0.0001.

bioRxiv preprint doi: https://doi.org/10.1101/2025.11.04.686414; this version posted November 5, 2025. The copyright holder for this preprint (which was not certified by peer review) is the author/funder, who has granted bioRxiv a license to display the preprint in perpetuity. It is made available under aCC-BY 4.0 International license.

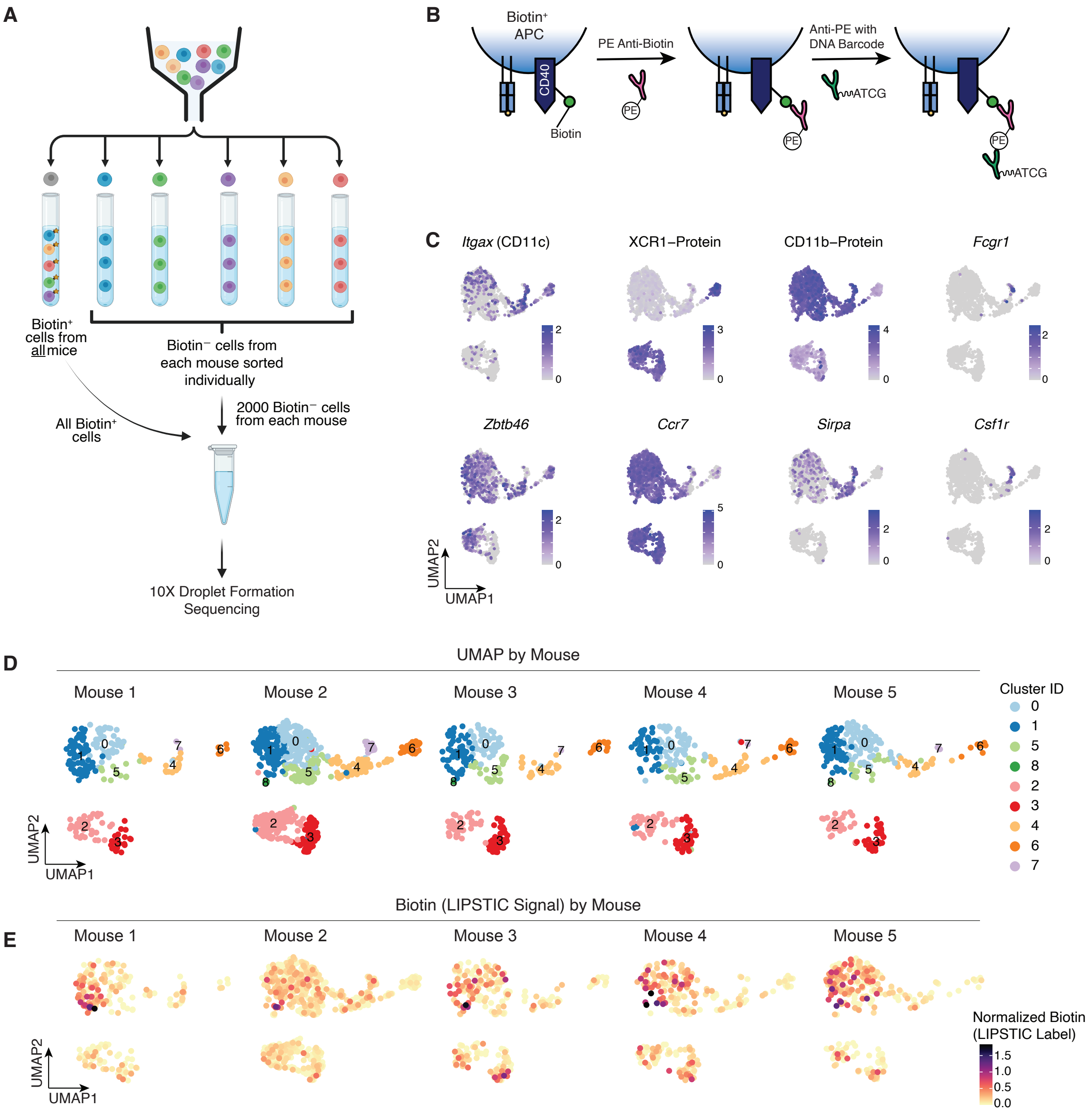

Supplementary Figure 5: Single cell RNA sequencing of sorted DCs from gLNs. (**A**) Schematic of sorting strategy for single cell sequencing of LIPSTIC labeled DCs. Biotin$^+$ DCs from all mice were sorted into one tube. Biotin$^-$ cells from each mouse were sorted individually. All biotin$^+$ cells were combined with a fraction of the biotin$^-$ cells from each mouse. (**B**) Schematic for how biotin (LIPSTIC label) was detected with Totalseq antibodies. Enriched DCs were first incubated with anti-biotin PE antibody so fluorescence could be used for sorting, then cells were incubated with the anti-PE TotalSeq antibody with a DNA barcode. (**C**) Feature plots showing the expression of key marker genes that were used to define cDC1s, cDC2s and migratory DCs clusters. Xcr1 and CD11b were detected at the protein level using TotalSeq antibodies. All other genes were detected at the RNA level. (**D**) Uniform manifold

bioRxiv preprint doi: https://doi.org/10.1101/2025.11.04.686414; this version posted November 5, 2025. The copyright holder for this preprint (which was not certified by peer review) is the author/funder, who has granted bioRxiv a license to display the preprint in perpetuity. It is made available under aCC-BY 4.0 International license.

approximation and projection (UMAP) plot of sorted DCs from gLNs in individual mice. All clusters contained cells from all mice. (**E**) Log normalized counts of LIPSTIC signal in individual mice. LIPSTIC signal detected via anti-PE hashtag antibody as outlined in (B).

bioRxiv preprint doi: https://doi.org/10.1101/2025.11.04.686414; this version posted November 5, 2025. The copyright holder for this preprint (which was not certified by peer review) is the author/funder, who has granted bioRxiv a license to display the preprint in perpetuity. It is made available under aCC-BY 4.0 International license.

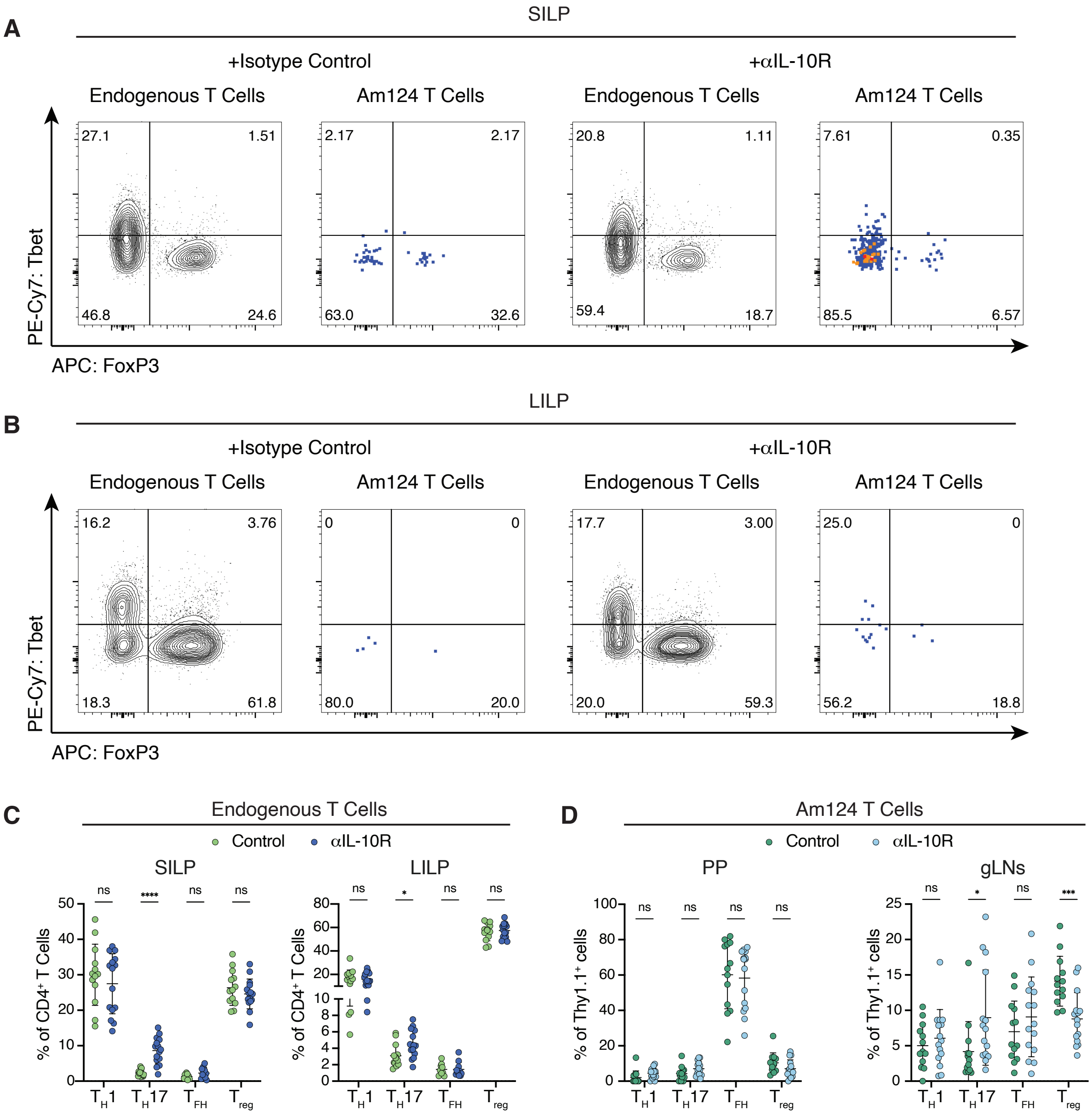

Supplementary Figure 6: Additional characterization of endogenous and Am124 T cells in αIL-10R-treated mice. (**A** and **B**) Representative flow plots showing expression of the T_H1 marker Tbet by endogenous and transferred T cells in the (**A**) SILP and (**B**) LILP of control mice and αIL-10R-treated mice. (**C**) Frequency of endogenous $CD4^+$ T cells expressing T_H1 ($Tbet^+$ $FoxP3^-$), T_H17 ($Ror\gamma t^+$ $FoxP3^-$), T_{FH} ($Bcl6^+$ $PD\text{-}1^+$) and T_{REG} ($FoxP3^+$) markers in the SILP (left) and LILP (right) of control and αIL-10R-treated mice. (**D**) Frequency of transferred Am124 T cells expressing T_H1 ($Tbet^+$ $FoxP3^-$), T_H17 ($Ror\gamma t^+$ $FoxP3^-$), T_{FH} ($Bcl6^+$ $PD\text{-}1^+$) and T_{REG} ($FoxP3^+$) markers in the PPs (left), and gLNs (right) of control and αIL-10R-treated mice. For all graphs, each symbol represents one mouse and the error bars represent mean and standard deviation; n = 4 to 6 mice per group, per experiment, data is pooled from three independent

bioRxiv preprint doi: https://doi.org/10.1101/2025.11.04.686414; this version posted November 5, 2025. The copyright holder for this preprint (which was not certified by peer review) is the author/funder, who has granted bioRxiv a license to display the preprint in perpetuity. It is made available under aCC-BY 4.0 International license.

experiments. P-values were calculated by unpaired T-test. Statistical significance denoted as not significant (ns), *P < 0.05, **P < 0.01, ***P < 0.001, ****P < 0.0001.

bioRxiv preprint doi: https://doi.org/10.1101/2025.11.04.686414; this version posted November 5, 2025. The copyright holder for this preprint (which was not certified by peer review) is the author/funder, who has granted bioRxiv a license to display the preprint in perpetuity. It is made available under aCC-BY 4.0 International license.

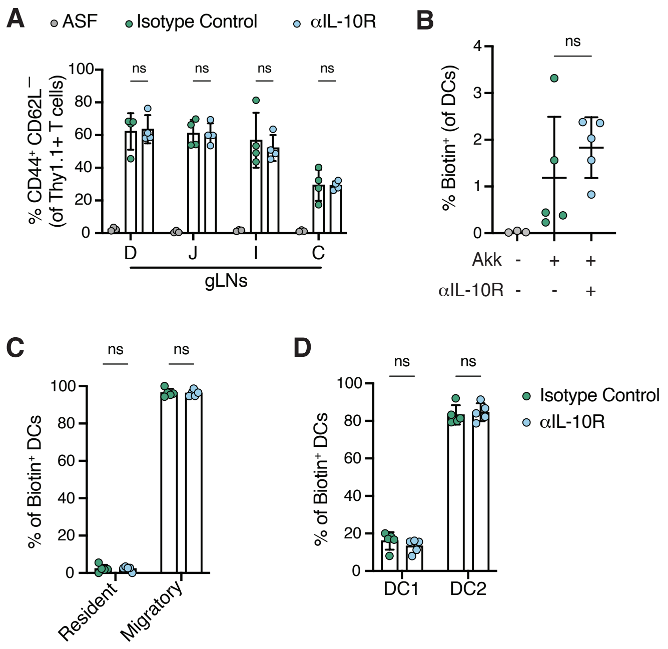

Supplementary Figure 7: αIL-10R treatment does not alter *A. muciniphila*-specific T cell priming in the gLNs or the identity of presenting APC. (**A**) $2.5x10^5$ Am124 T cells were adoptively transferred into ASF (untreated) and ASF+Akk mice 3 days after injection of 1 mg αIL-10R or isotype control antibodies. gLNs were harvested 2.5 days post-transfer. Percentage of activated ($CD44^+$ $CD62L^-$) Am124 T cells in gLNs of ASF and isotype- or αIL-10R-treated ASF+Akk mice (n = 3 to 4 per group, data is representative of two independent experiments). For (**B** to **D**), $1x10^6$ *Cd40lg*^{SrtAv2} Am124 T cells were adoptively transferred into ASF (untreated) and ASF+Akk mice 3 days after injection of 1 mg αIL-10R or isotype control antibodies. LIPSTIC labeling was performed 2.5 days later to identify *A. muciniphila*-presenting cells. (**B**) Percentage of LIPSTIC-labeled biotin$^+$ DCs in gLNs of ASF and isotype- or αIL-10R-treated ASF+Akk mice. (**C**) Percentage of migratory and resident cDCs out of total biotin$^+$ cDCs in ASF+Akk mice from (B). (**D**) Percentage of cDC1s (Xcr1$^+$) and cDC2s (Sirpα$^+$) cDCs out of total biotin$^+$ cDCs from ASF+Akk mice in (B). For (B to D), n = 3 to 5 mice per group, data is representative of three independent experiments. For all graphs, each symbol represents one mouse and error bars represent mean and standard deviation. P-values were calculated by unpaired T-test for (A), (C), and (D), and by one-way ANOVA for (B). Statistical significance denoted as not significant (ns), *P < 0.05, **P < 0.01, ***P < 0.001, ****P < 0.0001.

bioRxiv preprint doi: https://doi.org/10.1101/2025.11.04.686414; this version posted November 5, 2025. The copyright holder for this preprint (which was not certified by peer review) is the author/funder, who has granted bioRxiv a license to display the preprint in perpetuity. It is made available under aCC-BY 4.0 International license.

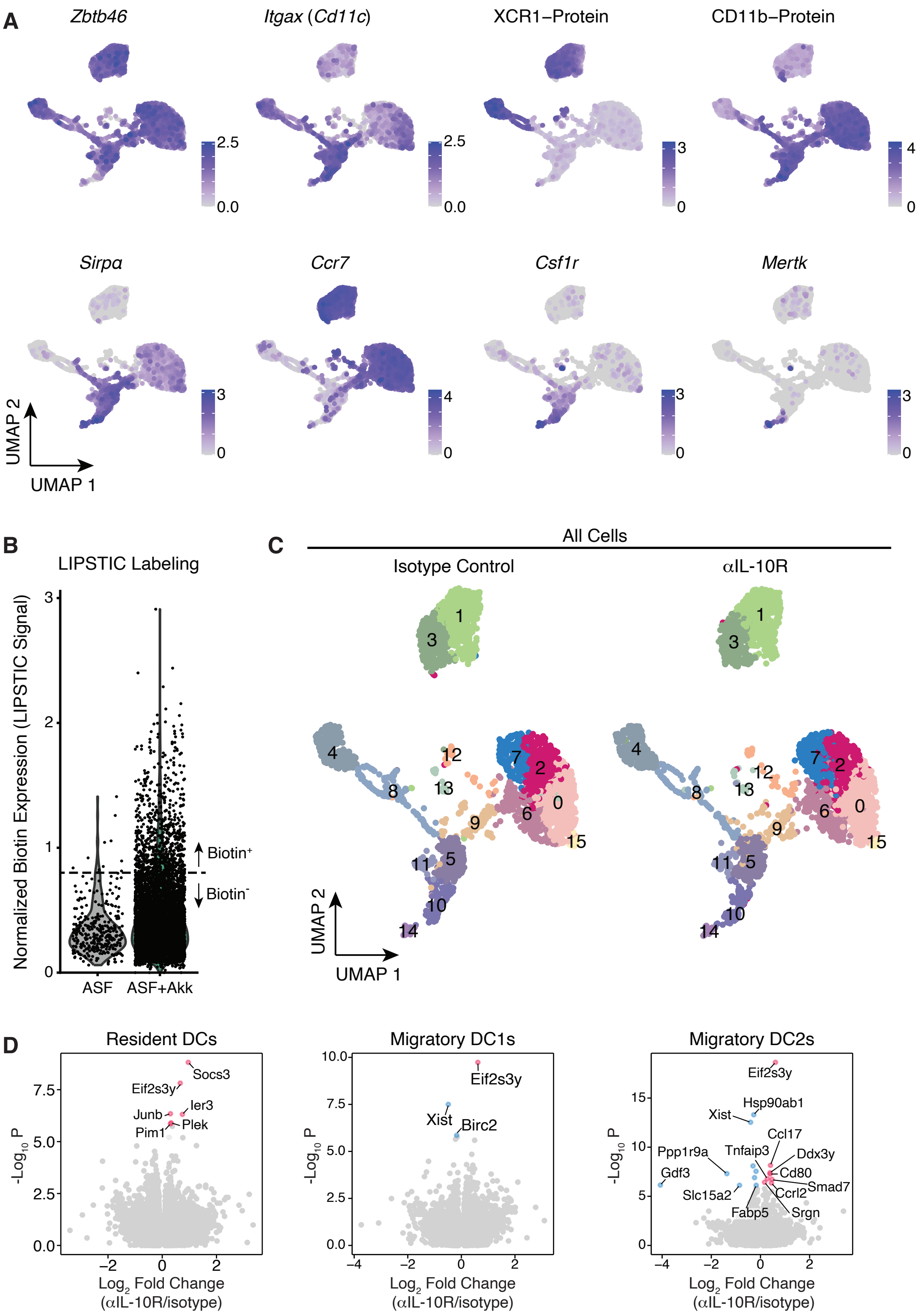

bioRxiv preprint doi: https://doi.org/10.1101/2025.11.04.686414; this version posted November 5, 2025. The copyright holder for this preprint (which was not certified by peer review) is the author/funder, who has granted bioRxiv a license to display the preprint in perpetuity. It is made available under aCC-BY 4.0 International license.

Supplementary Figure 8: Single cell RNA sequencing of sorted cDCs from gLNs of ASF+Akk mice treated with αIL-10R or isotype control antibody. (**A**) Feature plots showing the expression of key marker genes that were used to define cDC1, cDC2 and migratory cDCs clusters. Xcr1 and CD11b were detected at the protein level using TotalSeq antibodies. All other genes were detected at the RNA level. (**B**) Log normalized counts of LIPSTIC signal in cells from ASF and isotype- or αIL-10R-treated ASF+Akk mice detected via anti-PE hashtag antibody. Dashed line at 0.8 is the cutoff used in Fig. 4 for biotin^{+} cells. (**C**) Uniform manifold approximation and projection (UMAP) plot of sorted DCs from gLNs separated by treatment group. (**D**) Volcano plots showing differentially expressed genes between mice treated with αIL-10R and isotype control antibody for resident cDCs (left), migratory cDC1s (middle), and migratory cDC2s (right). Statistically significant genes are colored in red if they are enriched in αIL-10R-treated mice or blue if they are enriched in isotype control mice.

bioRxiv preprint doi: https://doi.org/10.1101/2025.11.04.686414; this version posted November 5, 2025. The copyright holder for this preprint (which was not certified by peer review) is the author/funder, who has granted bioRxiv a license to display the preprint in perpetuity. It is made available under aCC-BY 4.0 International license.

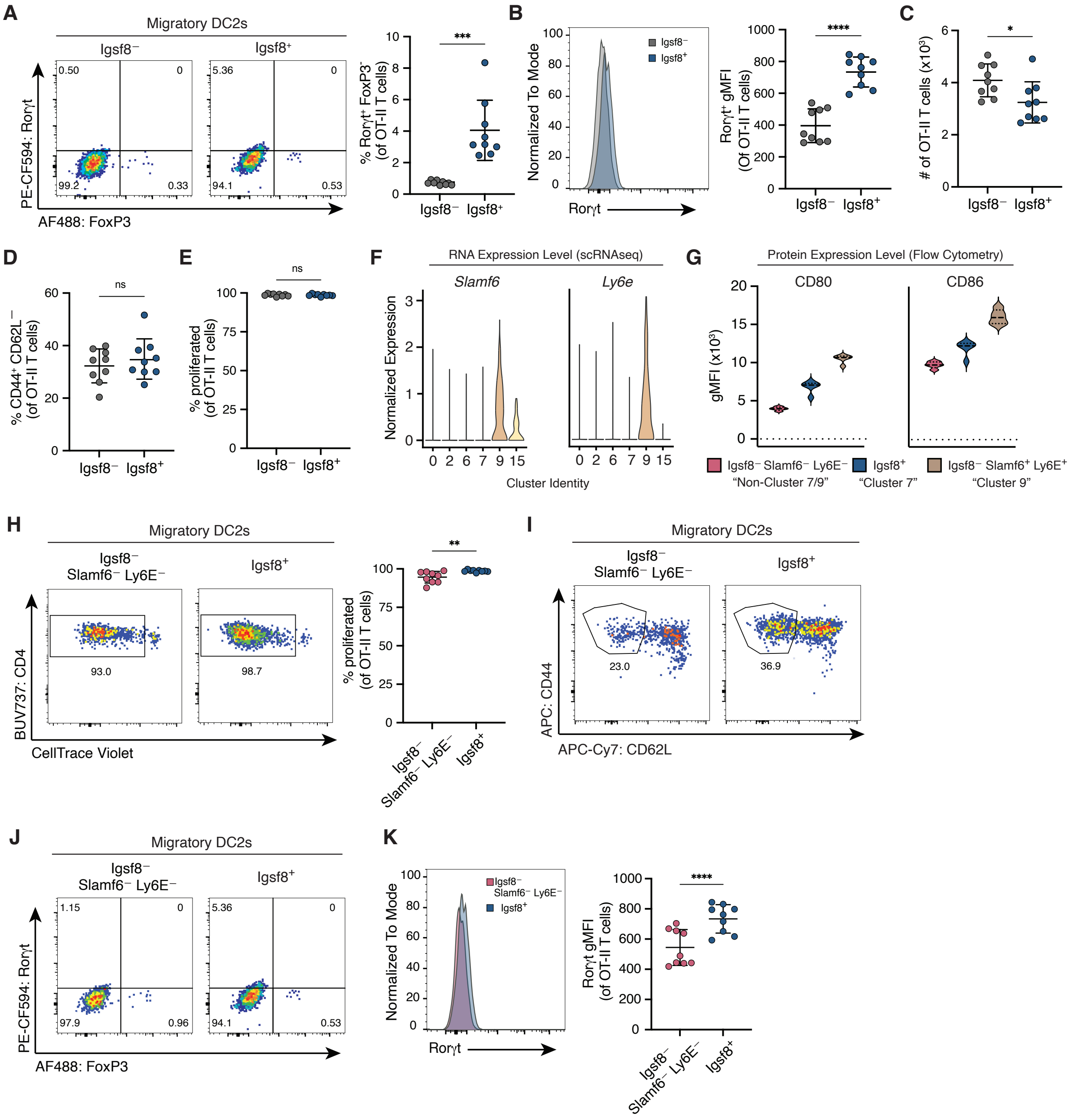

Supplementary Figure 9: Distinct migratory cDC2 subpopulations differentially promote T cell activation and T_H17 differentiation *in vitro*. For (**A** to **E**, and **H** to **K**), naive, CTV-labeled OT-II CD4⁺ T cells were co-cultured in the presence of exogenous OT-II peptide for 96 hours with the indicated migratory cDC2 populations sorted from the gLNs of untreated ASF+Akk mice. (**A**) Representative flow plots (left) and frequency (right) of Rorγt⁺ FoxP3⁻ OT-II T cells after co-culture with Igsf8⁻ or Igsf8⁺ migratory cDC2s. (**B**) Representative histograms (left) and quantification (right) of Rorγt expression in OT-II T cells after co-culture with Igsf8⁻ or Igsf8⁺ migratory cDC2s. (**C**) Total number of OT-II T cells after co-culture with Igsf8⁻ or Igsf8⁺ migratory cDC2s. (**D**) Frequency of activated (CD44⁺ CD62L⁻) OT-II cells after co-culture with

bioRxiv preprint doi: https://doi.org/10.1101/2025.11.04.686414; this version posted November 5, 2025. The copyright holder for this preprint (which was not certified by peer review) is the author/funder, who has granted bioRxiv a license to display the preprint in perpetuity. It is made available under aCC-BY 4.0 International license.

Igsf8$^{–}$ or Igsf8^{+} migratory cDC2s. (**E**) Frequency of proliferated OT-II cells after co-culture with Igsf8$^{–}$ or Igsf8^{+} migratory cDC2s. (**F**) Expression of the indicated genes at the RNA level in bulk migratory cDC2 clusters from scRNAseq experiment in Fig. 5. (**G**) Expression of the indicated genes at the protein level in Igsf8$^{–}$ Slamf6$^{–}$ Ly6E$^{–}$ ("non-cluster 7/9") Igsf8^{+} ("cluster 7) and Igsf8$^{–}$ Slamf6^{+} Ly6E^{+} ("cluster 9") migratory cDC2s. Solid and dashed lines indicate median and quartiles. n = 4 to 5 mice per experiment, data representative of two independent experiments. (**H**) Representative flow plots (left) and frequency (right) of proliferated OT-II T cells after co-culture with Igsf8$^{–}$ Slamf6$^{–}$ Ly6E$^{–}$ or Igsf8^{+} migratory cDC2s. (**I**) Representative flow plots of activated (CD44^{+} CD62L$^{–}$) OT-II T cells after co-culture with Igsf8$^{–}$ Slamf6$^{–}$ Ly6E$^{–}$ or Igsf8^{+} migratory cDC2s. (**J**) Representative flow plots of Rorγt^{+} FoxP3$^{–}$ OT-II T cells after co-culture with Igsf8$^{–}$ Slamf6$^{–}$ Ly6E$^{–}$ or Igsf8^{+} migratory cDC2s. (**K**) Representative histograms (left) and quantification (right) of Rorγt expression in OT-II T cells after co-culture with Igsf8$^{–}$ Slamf6$^{–}$ Ly6E$^{–}$ or Igsf8^{+} migratory cDC2s. For (A to E, and H to K), n = 4 to 5 mice per experiment; data pooled from two independent experiments. P-values were calculated via paired T-test. For all graphs, each symbol represents one mouse and error bars represent mean and standard deviation. Statistical significance denoted as not significant (ns), *P < 0.05, **P < 0.01, ***P < 0.001, ****P < 0.0001.

bioRxiv preprint doi: https://doi.org/10.1101/2025.11.04.686414; this version posted November 5, 2025. The copyright holder for this preprint (which was not certified by peer review) is the author/funder, who has granted bioRxiv a license to display the preprint in perpetuity. It is made available under aCC-BY 4.0 International license.

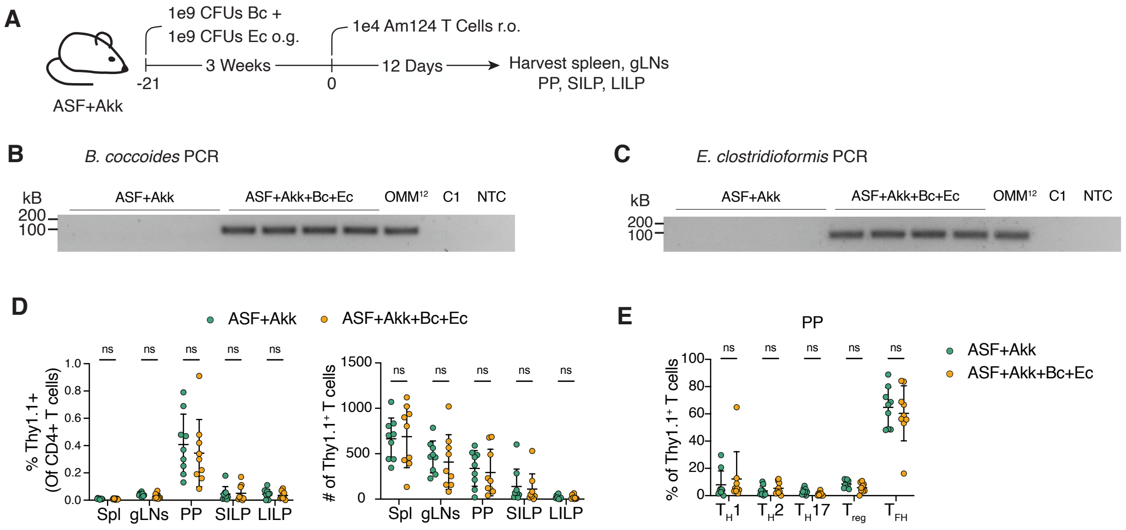

Supplementary Figure 10: Colonization with *B. coccoides* and *E. clostridioformis* does not alter the differentiation of *A. muciniphila*-specific T cells. (**A**) Schematic representation of experimental timeline. 1x10^9 CFUs of *B. coccoides* (Bc) and 1x10^9 CFUs of *E. clostridioformis* (Ec) were gavaged into ASF+Akk mice to make ASF+Akk+Bc+Ec mice. Three weeks post-gavage, 1x10^4 Am124 T cells were adoptively transferred into ASF+Akk and ASF+Akk+Bc+Ec mice. Spleen, gLNs, PPs, SILP, and LILP were harvested 12 days post-transfer. (**B** and **C**) Agarose gel of *B. coccoides*- (**B**) and *E. clostridioformis*- (**C**) specific amplicons from fecal DNA of indicated mice. Fecal DNA from OMM^{12} mice is used as a positive control. Two negative controls are included: a contamination control for the fecal prep (C1) and a non-template control (NTC). (**D**) Frequency (left) and number (right) of transferred Am124 T cells in tissues of ASF+Akk mice and ASF+Akk+Bc+Ec 12 days post-transfer. (**E**) Frequency of transferred Am124 T cells expressing T_H1 (Tbet$^+$ FoxP3$^-$), T_H2 (Gata3$^+$ FoxP3$^-$), T_H17 (Rorγt$^+$ FoxP3$^-$), T_{FH} (Bcl6$^+$ PD-1$^+$) and T_{REG} (FoxP3$^+$) markers in the PPs tissues of ASF+Akk mice and ASF+Akk+Bc+Ec. For (D and E), n = 4 to 5 mice per group, per experiment, data is pooled from two independent experiments. Each symbol represents one mouse and error bars represent mean and standard deviation. P-values were calculated using unpaired T-test. Statistical significance denoted as not significant (ns), *P < 0.05, **P < 0.01, ***P < 0.001, ****P < 0.0001.

bioRxiv preprint doi: https://doi.org/10.1101/2025.11.04.686414; this version posted November 5, 2025. The copyright holder for this preprint (which was not certified by peer review) is the author/funder, who has granted bioRxiv a license to display the preprint in perpetuity. It is made available under aCC-BY 4.0 International license.

Table S1. (separate file)

Primers and probes used for detecting bacterial species

Table S2. (separate file)

Antibodies and dyes used for flow cytometry

Table S3. (separate file)

Genes negative and positively correlated with biotin signal in migratory cDC2s in Fig. 3

Table S4. (separate file)

sc2markers output for cluster 7 migratory cDC2s in Fig. 5

Table S5. (separate file)

sc2markers output for cluster 9 migratory cDC2s in fig. S9
